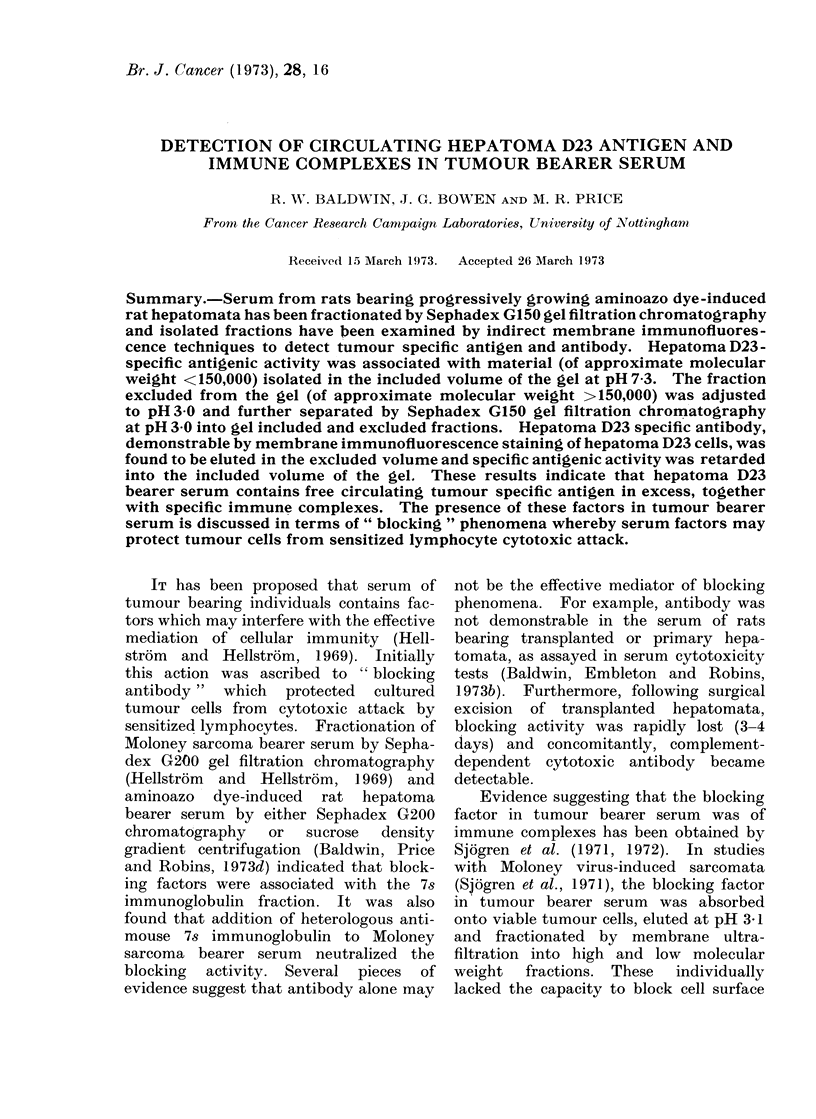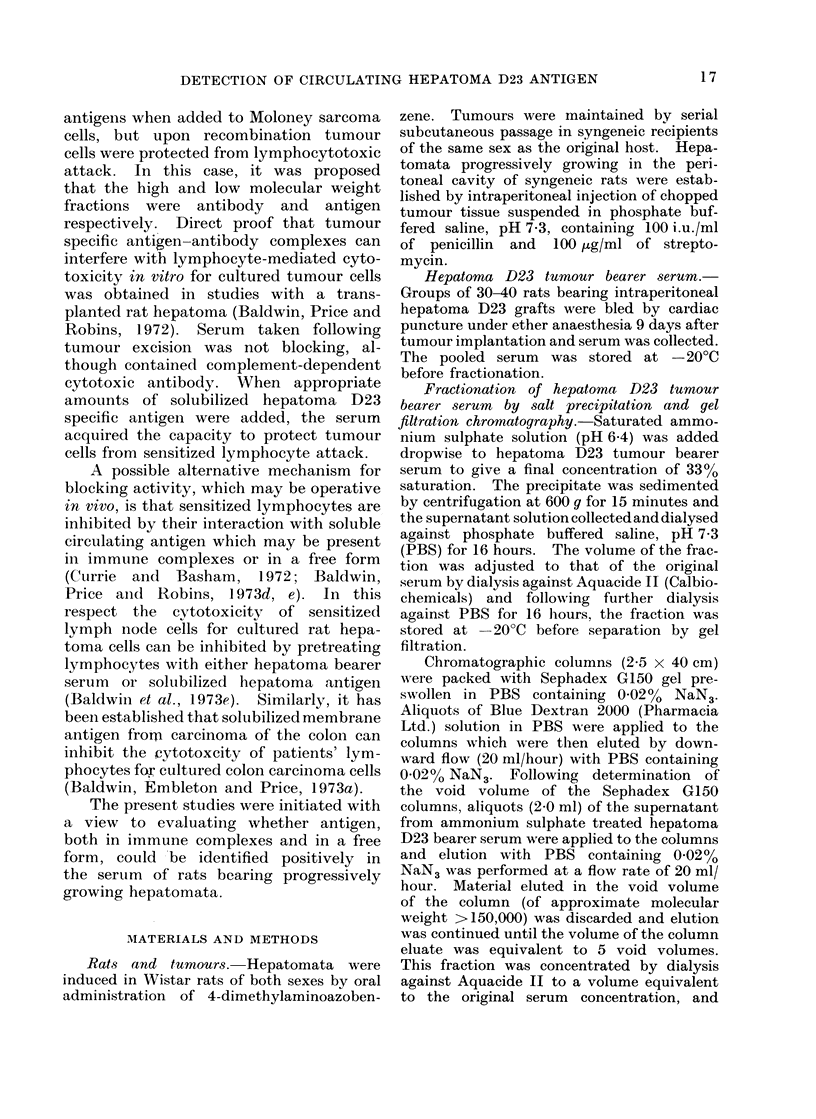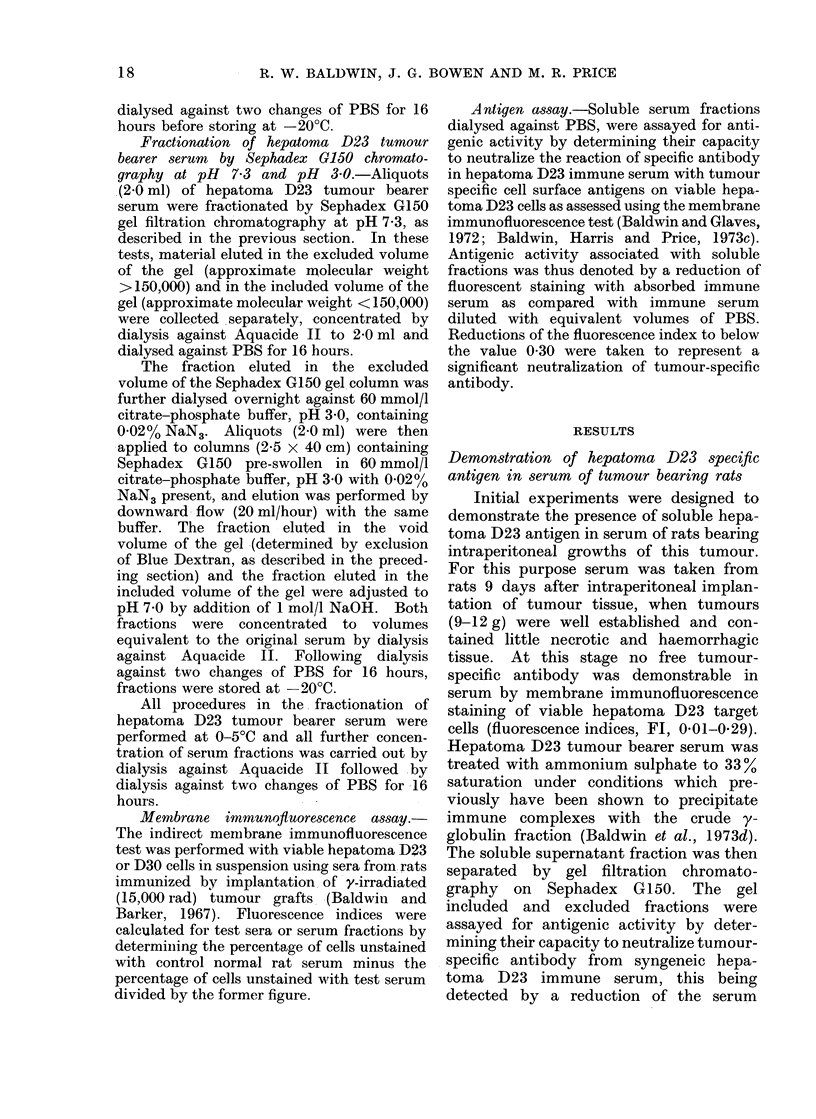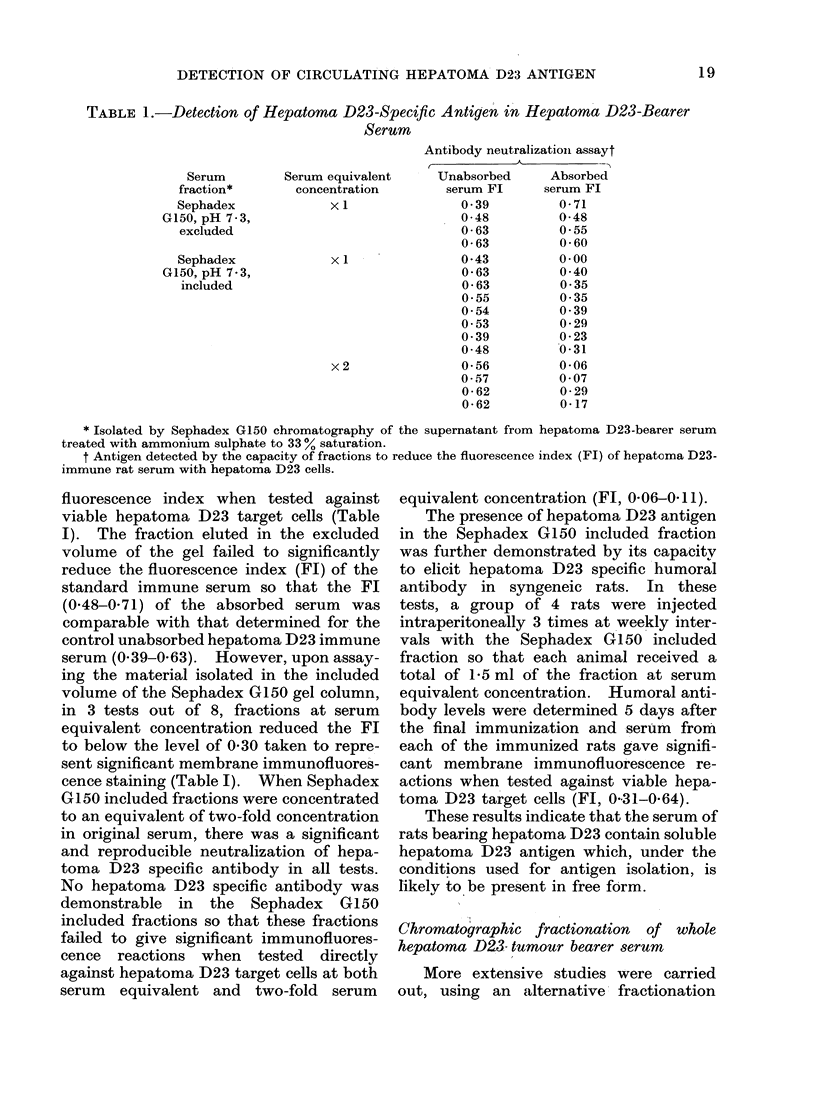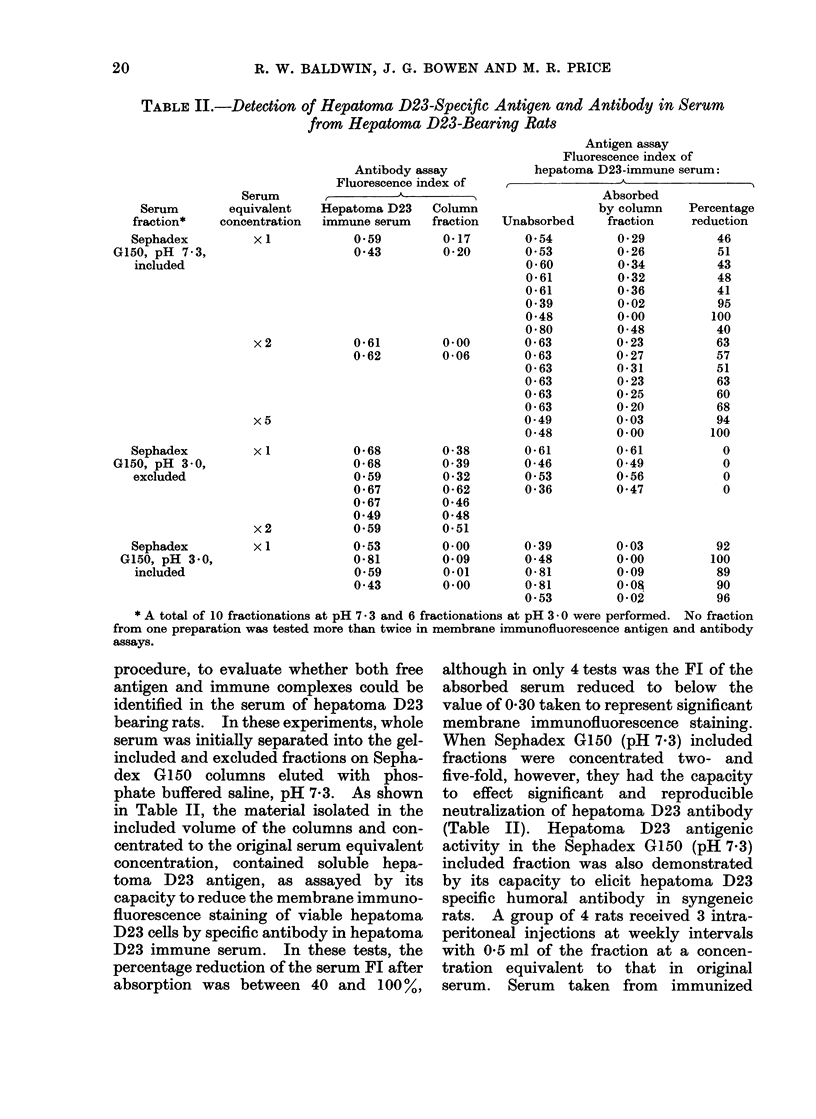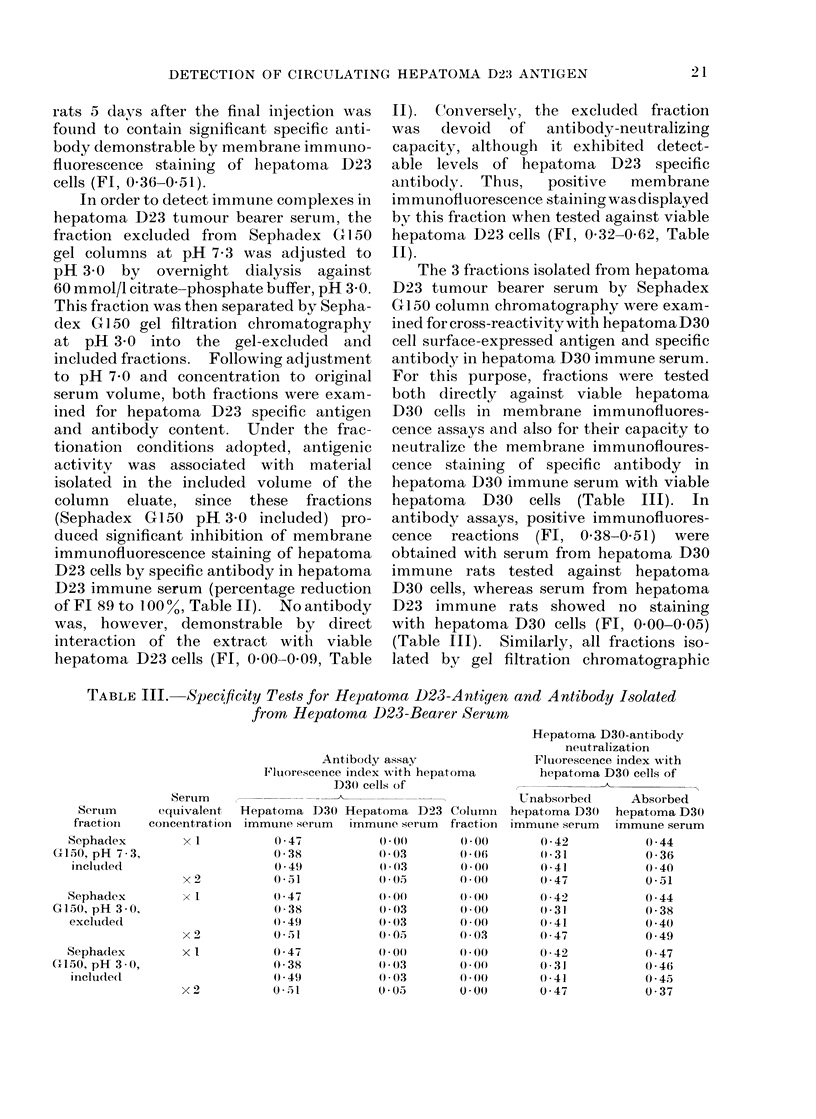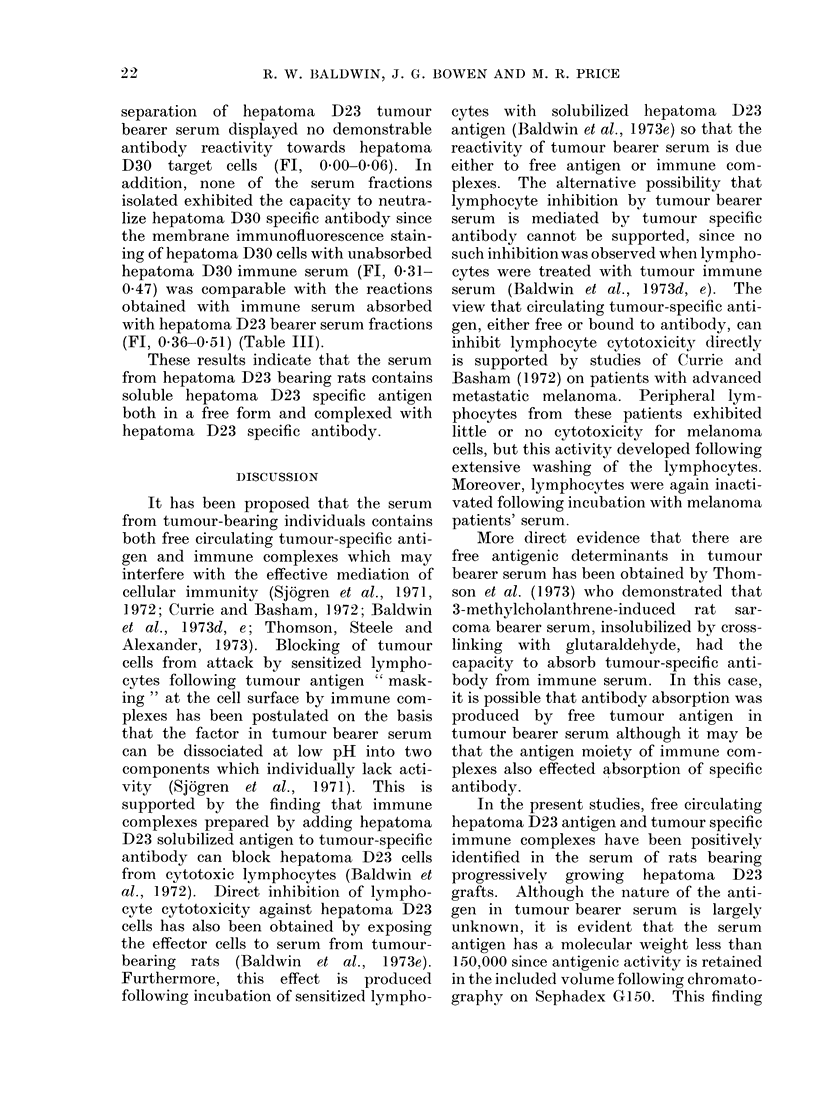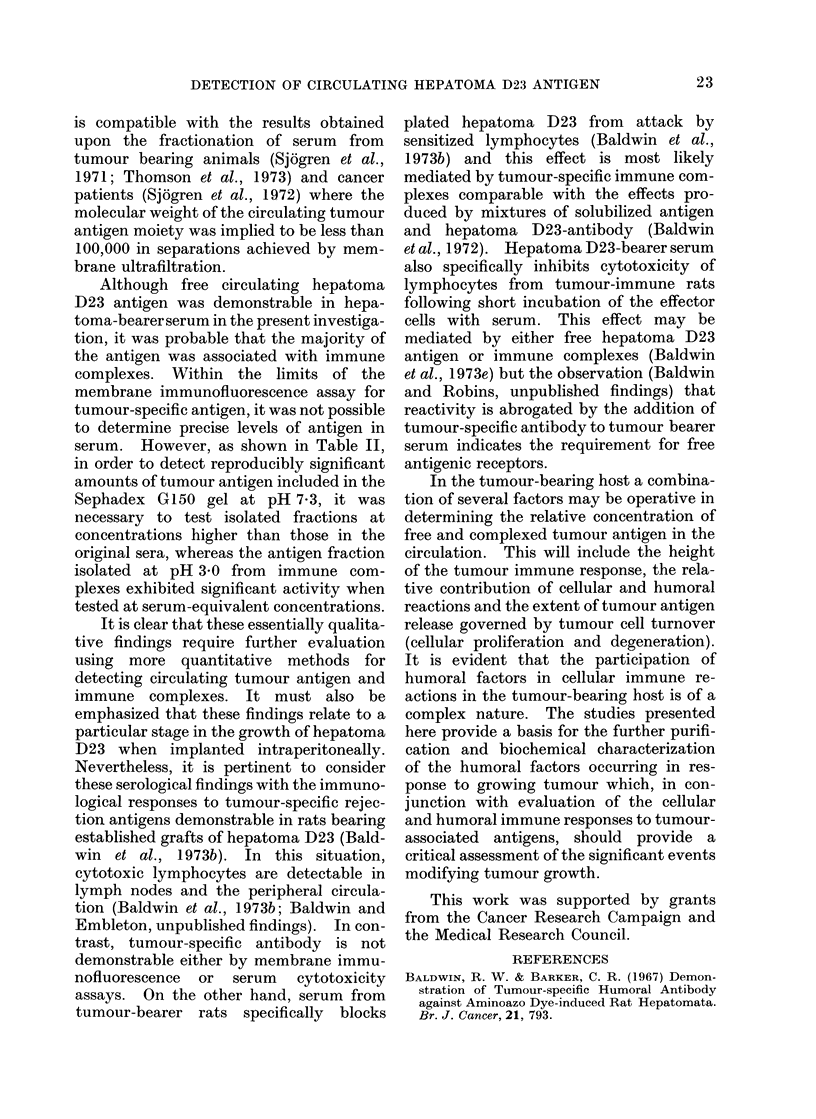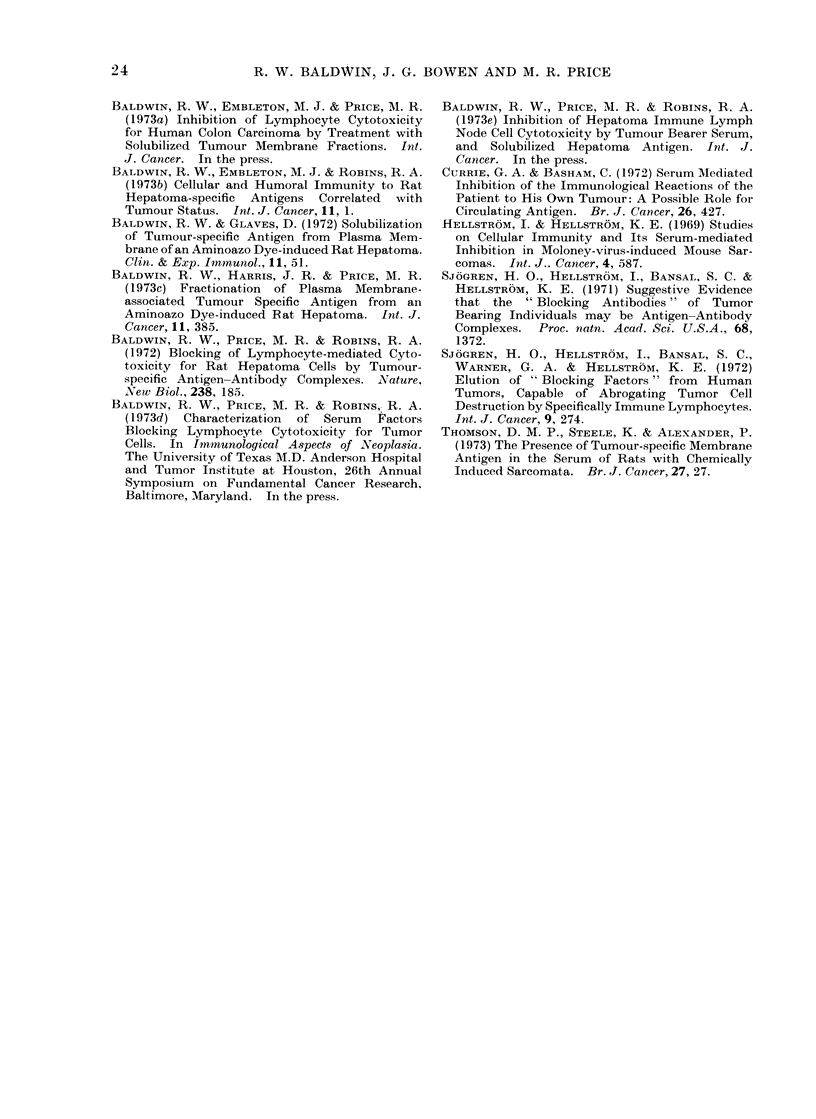# Detection of Circulating Hepatoma D23 Antigen and Immune Complexes in Tumour Bearer Serum

**DOI:** 10.1038/bjc.1973.66

**Published:** 1973-07

**Authors:** R. W. Baldwin, J. G. Bowen, M. R. Price

## Abstract

Serum from rats bearing progressively growing aminoazo dye-induced rat hepatomata has been fractionated by Sephadex G150 gel filtration chromatography and isolated fractions have been examined by indirect membrane immunofluorescence techniques to detect tumour specific antigen and antibody. Hepatoma D23-specific antigenic activity was associated with material (of approximate molecular weight <150,000) isolated in the included volume of the gel at pH 7·3. The fraction excluded from the gel (of approximate molecular weight >150,000) was adjusted to pH 3·0 and further separated by Sephadex G150 gel filtration chromatography at pH 3·0 into gel included and excluded fractions. Hepatoma D23 specific antibody, demonstrable by membrane immunofluorescence staining of hepatoma D23 cells, was found to be eluted in the excluded volume and specific antigenic activity was retarded into the included volume of the gel. These results indicate that hepatoma D23 bearer serum contains free circulating tumour specific antigen in excess, together with specific immune complexes. The presence of these factors in tumour bearer serum is discussed in terms of “blocking” phenomena whereby serum factors may protect tumour cells from sensitized lymphocyte cytotoxic attack.


*Br. J. Cancer* (1973), **28**, 16

### DETECTION OF CIRCULATING HEPATOMA D23 ANTIGEN AND IMMUNE COMPLEXES IN TUMOUR BEARER SERUM

R. W. BALDWIN, J. G. BOWEN AND M. R. PRICE

*From the Cancer Research Campaign Laboratories, University of Nottingham*

Received 15 March 1973. Accepted 26 March 1973

**Summary.—Serum from rats bearing progressively growing aminoazo dye-induced rat hepatomata has been fractionated by Sephadex G150 gel filtration chromatography and isolated fractions have been examined by indirect membrane immunofluorescence techniques to detect tumour specific antigen and antibody. Hepatoma D23-specific antigenic activity was associated with material (of approximate molecular weight <150,000) isolated in the included volume of the gel at pH 7·3. The fraction excluded from the gel (of approximate molecular weight >150,000) was adjusted to pH 3·0 and further separated by Sephadex G150 gel filtration chromatography at pH 3·0 into gel included and excluded fractions. Hepatoma D23 specific antibody, demonstrable by membrane immunofluorescence staining of hepatoma D23 cells, was found to be eluted in the excluded volume and specific antigenic activity was retarded into the included volume of the gel. These results indicate that hepatoma D23 bearer serum contains free circulating tumour specific antigen in excess, together with specific immune complexes. The presence of these factors in tumour bearer serum is discussed in terms of " blocking " phenomena whereby serum factors may protect tumour cells from sensitized lymphocyte cytotoxic attack.**

It has been proposed that serum of tumour bearing individuals contains factors which may interfere with the effective mediation of cellular immunity (Hellström and Hellström, 1969). Initially this action was ascribed to " blocking antibody " which protected cultured tumour cells from cytotoxic attack by sensitized lymphocytes. Fractionation of Moloney sarcoma bearer serum by Sephadex G200 gel filtration chromatography (Hellström and Hellström, 1969) and aminoazo dye-induced rat hepatoma bearer serum by either Sephadex G200 chromatography or sucrose density gradient centrifugation (Baldwin, Price and Robins, 1973*d*) indicated that blocking factors were associated with the 7*s* immunoglobulin fraction. It was also found that addition of heterologous anti-mouse 7*s* immunoglobulin to Moloney sarcoma bearer serum neutralized the blocking activity. Several pieces of evidence suggest that antibody alone may not be the effective mediator of blocking phenomena. For example, antibody was not demonstrable in the serum of rats bearing transplanted or primary hepatomata, as assayed in serum cytotoxicity tests (Baldwin, Embleton and Robins, 1973*b*). Furthermore, following surgical excision of transplanted hepatomata, blocking activity was rapidly lost (3–4 days) and concomitantly, complement-dependent cytotoxic antibody became detectable.

Evidence suggesting that the blocking factor in tumour bearer serum was of immune complexes has been obtained by Sjögren *et al.* (1971, 1972). In studies with Moloney virus-induced sarcomata (Sjögren *et al.*, 1971), the blocking factor in tumour bearer serum was absorbed onto viable tumour cells, eluted at pH 3·1 and fractionated by membrane ultrafiltration into high and low molecular weight fractions. These individually lacked the capacity to block cell surface antigens when added to Moloney sarcoma cells, but upon recombination tumour cells were protected from lymphocytotoxic attack. In this case, it was proposed that the high and low molecular weight fractions were antibody and antigen respectively. Direct proof that tumour specific antigen–antibody complexes can interfere with lymphocyte-mediated cytotoxicity *in vitro* for cultured tumour cells was obtained in studies with a transplanted rat hepatoma (Baldwin, Price and Robins, 1972). Serum taken following tumour excision was not blocking, although contained complement-dependent cytotoxic antibody. When appropriate amounts of solubilized hepatoma D23 specific antigen were added, the serum acquired the capacity to protect tumour cells from sensitized lymphocyte attack.

A possible alternative mechanism for blocking activity, which may be operative *in vivo*, is that sensitized lymphocytes are inhibited by their interaction with soluble circulating antigen which may be present in immune complexes or in a free form (Currie and Basham, 1972; Baldwin, Price and Robins, 1973*d*, *e*). In this respect the cytotoxicity of sensitized lymph node cells for cultured rat hepatoma cells can be inhibited by pretreating lymphocytes with either hepatoma bearer serum or solubilized hepatoma antigen (Baldwin *et al.*, 1973*e*). Similarly, it has been established that solubilized membrane antigen from carcinoma of the colon can inhibit the cytotoxcity of patients' lymphocytes for cultured colon carcinoma cells (Baldwin, Embleton and Price, 1973*a*).

The present studies were initiated with a view to evaluating whether antigen, both in immune complexes and in a free form, could be identified positively in the serum of rats bearing progressively growing hepatomata.

### MATERIALS AND METHODS

*Rats and tumours.*—Hepatomata were induced in Wistar rats of both sexes by oral administration of 4-dimethylaminoazobenzene. Tumours were maintained by serial subcutaneous passage in syngeneic recipients of the same sex as the original host. Hepatomata progressively growing in the peritoneal cavity of syngeneic rats were established by intraperitoneal injection of chopped tumour tissue suspended in phosphate buffered saline, pH 7·3, containing 100 i.u./ml of penicillin and 100 $\mu$g/ml of streptomycin.

*Hepatoma D23 tumour bearer serum.*—Groups of 30–40 rats bearing intraperitoneal hepatoma D23 grafts were bled by cardiac puncture under ether anaesthesia 9 days after tumour implantation and serum was collected. The pooled serum was stored at −20°C before fractionation.

*Fractionation of hepatoma D23 tumour bearer serum by salt precipitation and gel filtration chromatography.*—Saturated ammonium sulphate solution (pH 6·4) was added dropwise to hepatoma D23 tumour bearer serum to give a final concentration of 33% saturation. The precipitate was sedimented by centrifugation at 600 *g* for 15 minutes and the supernatant solution collected and dialysed against phosphate buffered saline, pH 7·3 (PBS) for 16 hours. The volume of the fraction was adjusted to that of the original serum by dialysis against Aquacide II (Calbiochemicals) and following further dialysis against PBS for 16 hours, the fraction was stored at −20°C before separation by gel filtration.

Chromatographic columns (2·5 × 40 cm) were packed with Sephadex G150 gel preswollen in PBS containing 0·02% $NaN_3$. Aliquots of Blue Dextran 2000 (Pharmacia Ltd.) solution in PBS were applied to the columns which were then eluted by downward flow (20 ml/hour) with PBS containing 0·02% $NaN_3$. Following determination of the void volume of the Sephadex G150 columns, aliquots (2·0 ml) of the supernatant from ammonium sulphate treated hepatoma D23 bearer serum were applied to the columns and elution with PBS containing 0·02% $NaN_3$ was performed at a flow rate of 20 ml/hour. Material eluted in the void volume of the column (of approximate molecular weight >150,000) was discarded and elution was continued until the volume of the column eluate was equivalent to 5 void volumes. This fraction was concentrated by dialysis against Aquacide II to a volume equivalent to the original serum concentration, and dialysed against two changes of PBS for 16 hours before storing at $-20°C$.

*Fractionation of hepatoma D23 tumour bearer serum by Sephadex G150 chromatography at pH 7·3 and pH 3·0.*—Aliquots (2·0 ml) of hepatoma D23 tumour bearer serum were fractionated by Sephadex G150 gel filtration chromatography at pH 7·3, as described in the previous section. In these tests, material eluted in the excluded volume of the gel (approximate molecular weight $>150{,}000$) and in the included volume of the gel (approximate molecular weight $<150{,}000$) were collected separately, concentrated by dialysis against Aquacide II to 2·0 ml and dialysed against PBS for 16 hours.

The fraction eluted in the excluded volume of the Sephadex G150 gel column was further dialysed overnight against 60 mmol/l citrate–phosphate buffer, pH 3·0, containing 0·02% $NaN_3$. Aliquots (2·0 ml) were then applied to columns ($2·5 \times 40$ cm) containing Sephadex G150 pre-swollen in 60 mmol/l citrate–phosphate buffer, pH 3·0 with 0·02% $NaN_3$ present, and elution was performed by downward flow (20 ml/hour) with the same buffer. The fraction eluted in the void volume of the gel (determined by exclusion of Blue Dextran, as described in the preceding section) and the fraction eluted in the included volume of the gel were adjusted to pH 7·0 by addition of 1 mol/l NaOH. Both fractions were concentrated to volumes equivalent to the original serum by dialysis against Aquacide II. Following dialysis against two changes of PBS for 16 hours, fractions were stored at $-20°C$.

All procedures in the fractionation of hepatoma D23 tumour bearer serum were performed at 0–5°C and all further concentration of serum fractions was carried out by dialysis against Aquacide II followed by dialysis against two changes of PBS for 16 hours.

*Membrane immunofluorescence assay.*—The indirect membrane immunofluorescence test was performed with viable hepatoma D23 or D30 cells in suspension using sera from rats immunized by implantation of $\gamma$-irradiated (15,000 rad) tumour grafts (Baldwin and Barker, 1967). Fluorescence indices were calculated for test sera or serum fractions by determining the percentage of cells unstained with control normal rat serum minus the percentage of cells unstained with test serum divided by the former figure.

*Antigen assay.*—Soluble serum fractions dialysed against PBS, were assayed for antigenic activity by determining their capacity to neutralize the reaction of specific antibody in hepatoma D23 immune serum with tumour specific cell surface antigens on viable hepatoma D23 cells as assessed using the membrane immunofluorescence test (Baldwin and Glaves, 1972; Baldwin, Harris and Price, 1973*c*). Antigenic activity associated with soluble fractions was thus denoted by a reduction of fluorescent staining with absorbed immune serum as compared with immune serum diluted with equivalent volumes of PBS. Reductions of the fluorescence index to below the value 0·30 were taken to represent a significant neutralization of tumour-specific antibody.

### RESULTS

*Demonstration of hepatoma D23 specific antigen in serum of tumour bearing rats*

Initial experiments were designed to demonstrate the presence of soluble hepatoma D23 antigen in serum of rats bearing intraperitoneal growths of this tumour. For this purpose serum was taken from rats 9 days after intraperitoneal implantation of tumour tissue, when tumours (9–12 g) were well established and contained little necrotic and haemorrhagic tissue. At this stage no free tumour-specific antibody was demonstrable in serum by membrane immunofluorescence staining of viable hepatoma D23 target cells (fluorescence indices, FI, 0·01–0·29). Hepatoma D23 tumour bearer serum was treated with ammonium sulphate to 33% saturation under conditions which previously have been shown to precipitate immune complexes with the crude $\gamma$-globulin fraction (Baldwin *et al.*, 1973*d*). The soluble supernatant fraction was then separated by gel filtration chromatography on Sephadex G150. The gel included and excluded fractions were assayed for antigenic activity by determining their capacity to neutralize tumour-specific antibody from syngeneic hepatoma D23 immune serum, this being detected by a reduction of the serum

TABLE 1.—*Detection of Hepatoma D23-Specific Antigen in Hepatoma D23-Bearer Serum*

| Serum fraction* | Serum equivalent concentration | Antibody neutralization assay† Unabsorbed serum FI | Absorbed serum FI |
|---|---|---|---|
| Sephadex G150, pH 7·3, excluded | ×1 | 0·39 | 0·71 |
| | | 0·48 | 0·48 |
| | | 0·63 | 0·55 |
| | | 0·63 | 0·60 |
| Sephadex G150, pH 7·3, included | ×1 | 0·43 | 0·00 |
| | | 0·63 | 0·40 |
| | | 0·63 | 0·35 |
| | | 0·55 | 0·35 |
| | | 0·54 | 0·39 |
| | | 0·53 | 0·29 |
| | | 0·39 | 0·23 |
| | | 0·48 | 0·31 |
| | ×2 | 0·56 | 0·06 |
| | | 0·57 | 0·07 |
| | | 0·62 | 0·29 |
| | | 0·62 | 0·17 |

* Isolated by Sephadex G150 chromatography of the supernatant from hepatoma D23-bearer serum treated with ammonium sulphate to 33% saturation.

† Antigen detected by the capacity of fractions to reduce the fluorescence index (FI) of hepatoma D23-immune rat serum with hepatoma D23 cells.

fluorescence index when tested against viable hepatoma D23 target cells (Table I). The fraction eluted in the excluded volume of the gel failed to significantly reduce the fluorescence index (FI) of the standard immune serum so that the FI (0·48–0·71) of the absorbed serum was comparable with that determined for the control unabsorbed hepatoma D23 immune serum (0·39–0·63). However, upon assaying the material isolated in the included volume of the Sephadex G150 gel column, in 3 tests out of 8, fractions at serum equivalent concentration reduced the FI to below the level of 0·30 taken to represent significant membrane immunofluorescence staining (Table I). When Sephadex G150 included fractions were concentrated to an equivalent of two-fold concentration in original serum, there was a significant and reproducible neutralization of hepatoma D23 specific antibody in all tests. No hepatoma D23 specific antibody was demonstrable in the Sephadex G150 included fractions so that these fractions failed to give significant immunofluorescence reactions when tested directly against hepatoma D23 target cells at both serum equivalent and two-fold serum equivalent concentration (FI, 0·06–0·11).

The presence of hepatoma D23 antigen in the Sephadex G150 included fraction was further demonstrated by its capacity to elicit hepatoma D23 specific humoral antibody in syngeneic rats. In these tests, a group of 4 rats were injected intraperitoneally 3 times at weekly intervals with the Sephadex G150 included fraction so that each animal received a total of 1·5 ml of the fraction at serum equivalent concentration. Humoral antibody levels were determined 5 days after the final immunization and serum from each of the immunized rats gave significant membrane immunofluorescence reactions when tested against viable hepatoma D23 target cells (FI, 0·31–0·64).

These results indicate that the serum of rats bearing hepatoma D23 contain soluble hepatoma D23 antigen which, under the conditions used for antigen isolation, is likely to be present in free form.

### *Chromatographic fractionation of whole hepatoma D23 tumour bearer serum*

More extensive studies were carried out, using an alternative fractionation

TABLE II.—*Detection of Hepatoma D23-Specific Antigen and Antibody in Serum from Hepatoma D23-Bearing Rats*

| | | Antibody assay Fluorescence index of | | Antigen assay Fluorescence index of hepatoma D23-immune serum: | | |
|---|---|---|---|---|---|---|
| Serum fraction* | Serum equivalent concentration | Hepatoma D23 immune serum | Column fraction | Unabsorbed | Absorbed by column fraction | Percentage reduction |
| Sephadex G150, pH 7·3, included | ×1 | 0·59 | 0·17 | 0·54 | 0·29 | 46 |
| | | 0·43 | 0·20 | 0·53 | 0·26 | 51 |
| | | | | 0·60 | 0·34 | 43 |
| | | | | 0·61 | 0·32 | 48 |
| | | | | 0·61 | 0·36 | 41 |
| | | | | 0·39 | 0·02 | 95 |
| | | | | 0·48 | 0·00 | 100 |
| | | | | 0·80 | 0·48 | 40 |
| | ×2 | 0·61 | 0·00 | 0·63 | 0·23 | 63 |
| | | 0·62 | 0·06 | 0·63 | 0·27 | 57 |
| | | | | 0·63 | 0·31 | 51 |
| | | | | 0·63 | 0·23 | 63 |
| | | | | 0·63 | 0·25 | 60 |
| | | | | 0·63 | 0·20 | 68 |
| | ×5 | | | 0·49 | 0·03 | 94 |
| | | | | 0·48 | 0·00 | 100 |
| Sephadex G150, pH 3·0, excluded | ×1 | 0·68 | 0·38 | 0·61 | 0·61 | 0 |
| | | 0·68 | 0·39 | 0·46 | 0·49 | 0 |
| | | 0·59 | 0·32 | 0·53 | 0·56 | 0 |
| | | 0·67 | 0·62 | 0·36 | 0·47 | 0 |
| | | 0·67 | 0·46 | | | |
| | | 0·49 | 0·48 | | | |
| | ×2 | 0·59 | 0·51 | | | |
| Sephadex G150, pH 3·0, included | ×1 | 0·53 | 0·00 | 0·39 | 0·03 | 92 |
| | | 0·81 | 0·09 | 0·48 | 0·00 | 100 |
| | | 0·59 | 0·01 | 0·81 | 0·09 | 89 |
| | | 0·43 | 0·00 | 0·81 | 0·08 | 90 |
| | | | | 0·53 | 0·02 | 96 |

* A total of 10 fractionations at pH 7·3 and 6 fractionations at pH 3·0 were performed. No fraction from one preparation was tested more than twice in membrane immunofluorescence antigen and antibody assays.

procedure, to evaluate whether both free antigen and immune complexes could be identified in the serum of hepatoma D23 bearing rats. In these experiments, whole serum was initially separated into the gel-included and excluded fractions on Sephadex G150 columns eluted with phosphate buffered saline, pH 7·3. As shown in Table II, the material isolated in the included volume of the columns and concentrated to the original serum equivalent concentration, contained soluble hepatoma D23 antigen, as assayed by its capacity to reduce the membrane immunofluorescence staining of viable hepatoma D23 cells by specific antibody in hepatoma D23 immune serum. In these tests, the percentage reduction of the serum FI after absorption was between 40 and 100%, although in only 4 tests was the FI of the absorbed serum reduced to below the value of 0·30 taken to represent significant membrane immunofluorescence staining. When Sephadex G150 (pH 7·3) included fractions were concentrated two- and five-fold, however, they had the capacity to effect significant and reproducible neutralization of hepatoma D23 antibody (Table II). Hepatoma D23 antigenic activity in the Sephadex G150 (pH 7·3) included fraction was also demonstrated by its capacity to elicit hepatoma D23 specific humoral antibody in syngeneic rats. A group of 4 rats received 3 intraperitoneal injections at weekly intervals with 0·5 ml of the fraction at a concentration equivalent to that in original serum. Serum taken from immunized rats 5 days after the final injection was found to contain significant specific antibody demonstrable by membrane immunofluorescence staining of hepatoma D23 cells (FI, 0·36–0·51).

In order to detect immune complexes in hepatoma D23 tumour bearer serum, the fraction excluded from Sephadex G150 gel columns at pH 7·3 was adjusted to pH 3·0 by overnight dialysis against 60 mmol/l citrate–phosphate buffer, pH 3·0. This fraction was then separated by Sephadex G150 gel filtration chromatography at pH 3·0 into the gel-excluded and included fractions. Following adjustment to pH 7·0 and concentration to original serum volume, both fractions were examined for hepatoma D23 specific antigen and antibody content. Under the fractionation conditions adopted, antigenic activity was associated with material isolated in the included volume of the column eluate, since these fractions (Sephadex G150 pH 3·0 included) produced significant inhibition of membrane immunofluorescence staining of hepatoma D23 cells by specific antibody in hepatoma D23 immune serum (percentage reduction of FI 89 to 100%, Table II). No antibody was, however, demonstrable by direct interaction of the extract with viable hepatoma D23 cells (FI, 0·00–0·09, Table II). Conversely, the excluded fraction was devoid of antibody-neutralizing capacity, although it exhibited detectable levels of hepatoma D23 specific antibody. Thus, positive membrane immunofluorescence staining was displayed by this fraction when tested against viable hepatoma D23 cells (FI, 0·32–0·62, Table II).

The 3 fractions isolated from hepatoma D23 tumour bearer serum by Sephadex G150 column chromatography were examined for cross-reactivity with hepatoma D30 cell surface-expressed antigen and specific antibody in hepatoma D30 immune serum. For this purpose, fractions were tested both directly against viable hepatoma D30 cells in membrane immunofluorescence assays and also for their capacity to neutralize the membrane immunoflourescence staining of specific antibody in hepatoma D30 immune serum with viable hepatoma D30 cells (Table III). In antibody assays, positive immunofluorescence reactions (FI, 0·38–0·51) were obtained with serum from hepatoma D30 immune rats tested against hepatoma D30 cells, whereas serum from hepatoma D23 immune rats showed no staining with hepatoma D30 cells (FI, 0·00–0·05) (Table III). Similarly, all fractions isolated by gel filtration chromatographic

TABLE III.—*Specificity Tests for Hepatoma D23-Antigen and Antibody Isolated from Hepatoma D23-Bearer Serum*

| Serum fraction | Serum equivalent concentration | Antibody assay Fluorescence index with hepatoma D30 cells of: Hepatoma D30 immune serum | Hepatoma D23 immune serum | Column fraction | Hepatoma D30-antibody neutralization Fluorescence index with hepatoma D30 cells of: Unabsorbed hepatoma D30 immune serum | Absorbed hepatoma D30 immune serum |
|---|---|---|---|---|---|---|
| Sephadex G150, pH 7·3, included | ×1 | 0·47 | 0·00 | 0·00 | 0·42 | 0·44 |
| | | 0·38 | 0·03 | 0·06 | 0·31 | 0·36 |
| | | 0·49 | 0·03 | 0·00 | 0·41 | 0·40 |
| | ×2 | 0·51 | 0·05 | 0·00 | 0·47 | 0·51 |
| Sephadex G150, pH 3·0, excluded | ×1 | 0·47 | 0·00 | 0·00 | 0·42 | 0·44 |
| | | 0·38 | 0·03 | 0·00 | 0·31 | 0·38 |
| | | 0·49 | 0·03 | 0·00 | 0·41 | 0·40 |
| | ×2 | 0·51 | 0·05 | 0·03 | 0·47 | 0·49 |
| Sephadex G150, pH 3·0, included | ×1 | 0·47 | 0·00 | 0·00 | 0·42 | 0·47 |
| | | 0·38 | 0·03 | 0·00 | 0·31 | 0·46 |
| | | 0·49 | 0·03 | 0·00 | 0·41 | 0·45 |
| | ×2 | 0·51 | 0·05 | 0·00 | 0·47 | 0·37 |

separation of hepatoma D23 tumour bearer serum displayed no demonstrable antibody reactivity towards hepatoma D30 target cells (FI, 0·00–0·06). In addition, none of the serum fractions isolated exhibited the capacity to neutralize hepatoma D30 specific antibody since the membrane immunofluorescence staining of hepatoma D30 cells with unabsorbed hepatoma D30 immune serum (FI, 0·31–0·47) was comparable with the reactions obtained with immune serum absorbed with hepatoma D23 bearer serum fractions (FI, 0·36–0·51) (Table III).

These results indicate that the serum from hepatoma D23 bearing rats contains soluble hepatoma D23 specific antigen both in a free form and complexed with hepatoma D23 specific antibody.

### DISCUSSION

It has been proposed that the serum from tumour-bearing individuals contains both free circulating tumour-specific antigen and immune complexes which may interfere with the effective mediation of cellular immunity (Sjögren *et al.*, 1971, 1972; Currie and Basham, 1972; Baldwin *et al.*, 1973*d*, *e*; Thomson, Steele and Alexander, 1973). Blocking of tumour cells from attack by sensitized lymphocytes following tumour antigen " masking " at the cell surface by immune complexes has been postulated on the basis that the factor in tumour bearer serum can be dissociated at low pH into two components which individually lack activity (Sjögren *et al.*, 1971). This is supported by the finding that immune complexes prepared by adding hepatoma D23 solubilized antigen to tumour-specific antibody can block hepatoma D23 cells from cytotoxic lymphocytes (Baldwin *et al.*, 1972). Direct inhibition of lymphocyte cytotoxicity against hepatoma D23 cells has also been obtained by exposing the effector cells to serum from tumour-bearing rats (Baldwin *et al.*, 1973*e*). Furthermore, this effect is produced following incubation of sensitized lymphocytes with solubilized hepatoma D23 antigen (Baldwin *et al.*, 1973*e*) so that the reactivity of tumour bearer serum is due either to free antigen or immune complexes. The alternative possibility that lymphocyte inhibition by tumour bearer serum is mediated by tumour specific antibody cannot be supported, since no such inhibition was observed when lymphocytes were treated with tumour immune serum (Baldwin *et al.*, 1973*d*, *e*). The view that circulating tumour-specific antigen, either free or bound to antibody, can inhibit lymphocyte cytotoxicity directly is supported by studies of Currie and Basham (1972) on patients with advanced metastatic melanoma. Peripheral lymphocytes from these patients exhibited little or no cytotoxicity for melanoma cells, but this activity developed following extensive washing of the lymphocytes. Moreover, lymphocytes were again inactivated following incubation with melanoma patients' serum.

More direct evidence that there are free antigenic determinants in tumour bearer serum has been obtained by Thomson *et al.* (1973) who demonstrated that 3-methylcholanthrene-induced rat sarcoma bearer serum, insolubilized by cross-linking with glutaraldehyde, had the capacity to absorb tumour-specific antibody from immune serum. In this case, it is possible that antibody absorption was produced by free tumour antigen in tumour bearer serum although it may be that the antigen moiety of immune complexes also effected absorption of specific antibody.

In the present studies, free circulating hepatoma D23 antigen and tumour specific immune complexes have been positively identified in the serum of rats bearing progressively growing hepatoma D23 grafts. Although the nature of the antigen in tumour bearer serum is largely unknown, it is evident that the serum antigen has a molecular weight less than 150,000 since antigenic activity is retained in the included volume following chromatography on Sephadex G150. This finding is compatible with the results obtained upon the fractionation of serum from tumour bearing animals (Sjögren *et al.*, 1971; Thomson *et al.*, 1973) and cancer patients (Sjögren *et al.*, 1972) where the molecular weight of the circulating tumour antigen moiety was implied to be less than 100,000 in separations achieved by membrane ultrafiltration.

Although free circulating hepatoma D23 antigen was demonstrable in hepatoma-bearer serum in the present investigation, it was probable that the majority of the antigen was associated with immune complexes. Within the limits of the membrane immunofluorescence assay for tumour-specific antigen, it was not possible to determine precise levels of antigen in serum. However, as shown in Table II, in order to detect reproducibly significant amounts of tumour antigen included in the Sephadex G150 gel at pH 7·3, it was necessary to test isolated fractions at concentrations higher than those in the original sera, whereas the antigen fraction isolated at pH 3·0 from immune complexes exhibited significant activity when tested at serum-equivalent concentrations.

It is clear that these essentially qualitative findings require further evaluation using more quantitative methods for detecting circulating tumour antigen and immune complexes. It must also be emphasized that these findings relate to a particular stage in the growth of hepatoma D23 when implanted intraperitoneally. Nevertheless, it is pertinent to consider these serological findings with the immunological responses to tumour-specific rejection antigens demonstrable in rats bearing established grafts of hepatoma D23 (Baldwin *et al.*, 1973*b*). In this situation, cytotoxic lymphocytes are detectable in lymph nodes and the peripheral circulation (Baldwin *et al.*, 1973*b*; Baldwin and Embleton, unpublished findings). In contrast, tumour-specific antibody is not demonstrable either by membrane immunofluorescence or serum cytotoxicity assays. On the other hand, serum from tumour-bearer rats specifically blocks plated hepatoma D23 from attack by sensitized lymphocytes (Baldwin *et al.*, 1973*b*) and this effect is most likely mediated by tumour-specific immune complexes comparable with the effects produced by mixtures of solubilized antigen and hepatoma D23-antibody (Baldwin *et al.*, 1972). Hepatoma D23-bearer serum also specifically inhibits cytotoxicity of lymphocytes from tumour-immune rats following short incubation of the effector cells with serum. This effect may be mediated by either free hepatoma D23 antigen or immune complexes (Baldwin *et al.*, 1973*e*) but the observation (Baldwin and Robins, unpublished findings) that reactivity is abrogated by the addition of tumour-specific antibody to tumour bearer serum indicates the requirement for free antigenic receptors.

In the tumour-bearing host a combination of several factors may be operative in determining the relative concentration of free and complexed tumour antigen in the circulation. This will include the height of the tumour immune response, the relative contribution of cellular and humoral reactions and the extent of tumour antigen release governed by tumour cell turnover (cellular proliferation and degeneration). It is evident that the participation of humoral factors in cellular immune reactions in the tumour-bearing host is of a complex nature. The studies presented here provide a basis for the further purification and biochemical characterization of the humoral factors occurring in response to growing tumour which, in conjunction with evaluation of the cellular and humoral immune responses to tumour-associated antigens, should provide a critical assessment of the significant events modifying tumour growth.

This work was supported by grants from the Cancer Research Campaign and the Medical Research Council.

### REFERENCES

BALDWIN, R. W. & BARKER, C. R. (1967) Demonstration of Tumour-specific Humoral Antibody against Aminoazo Dye-induced Rat Hepatomata. *Br. J. Cancer*, **21**, 793.

BALDWIN, R. W., EMBLETON, M. J. & PRICE, M. R. (1973*a*) Inhibition of Lymphocyte Cytotoxicity for Human Colon Carcinoma by Treatment with Solubilized Tumour Membrane Fractions. *Int. J. Cancer*. In the press.

BALDWIN, R. W., EMBLETON, M. J. & ROBINS, R. A. (1973*b*) Cellular and Humoral Immunity to Rat Hepatoma-specific Antigens Correlated with Tumour Status. *Int. J. Cancer*, **11**, 1.

BALDWIN, R. W. & GLAVES, D. (1972) Solubilization of Tumour-specific Antigen from Plasma Membrane of an Aminoazo Dye-induced Rat Hepatoma. *Clin. & Exp. Immunol.*, **11**, 51.

BALDWIN, R. W., HARRIS, J. R. & PRICE, M. R. (1973*c*) Fractionation of Plasma Membrane-associated Tumour Specific Antigen from an Aminoazo Dye-induced Rat Hepatoma. *Int. J. Cancer*, **11**, 385.

BALDWIN, R. W., PRICE, M. R. & ROBINS, R. A. (1972) Blocking of Lymphocyte-mediated Cytotoxicity for Rat Hepatoma Cells by Tumour-specific Antigen–Antibody Complexes. *Nature, New Biol.*, **238**, 185.

BALDWIN, R. W., PRICE, M. R. & ROBINS, R. A. (1973*d*) Characterization of Serum Factors Blocking Lymphocyte Cytotoxicity for Tumor Cells. In *Immunological Aspects of Neoplasia*. The University of Texas M.D. Anderson Hospital and Tumor Institute at Houston, 26th Annual Symposium on Fundamental Cancer Research, Baltimore, Maryland. In the press.

BALDWIN, R. W., PRICE, M. R. & ROBINS, R. A. (1973*e*) Inhibition of Hepatoma Immune Lymph Node Cell Cytotoxicity by Tumour Bearer Serum, and Solubilized Hepatoma Antigen. *Int. J. Cancer*. In the press.

CURRIE, G. A. & BASHAM, C. (1972) Serum Mediated Inhibition of the Immunological Reactions of the Patient to His Own Tumour: A Possible Role for Circulating Antigen. *Br. J. Cancer*, **26**, 427.

HELLSTRÖM, I. & HELLSTRÖM, K. E. (1969) Studies on Cellular Immunity and Its Serum-mediated Inhibition in Moloney-virus-induced Mouse Sarcomas. *Int. J.. Cancer*, **4**, 587.

SJÖGREN, H. O., HELLSTRÖM, I., BANSAL, S. C. & HELLSTRÖM, K. E. (1971) Suggestive Evidence that the "Blocking Antibodies" of Tumor Bearing Individuals may be Antigen–Antibody Complexes. *Proc. natn. Acad. Sci. U.S.A.*, **68**, 1372.

SJÖGREN, H. O., HELLSTRÖM, I., BANSAL, S. C., WARNER, G. A. & HELLSTRÖM, K. E. (1972) Elution of "Blocking Factors" from Human Tumors, Capable of Abrogating Tumor Cell Destruction by Specifically Immune Lymphocytes. *Int. J. Cancer*, **9**, 274.

THOMSON, D. M. P., STEELE, K. & ALEXANDER, P. (1973) The Presence of Tumour-specific Membrane Antigen in the Serum of Rats with Chemically Induced Sarcomata. *Br. J. Cancer*, **27**, 27.